# Achieving Temperature‐Insensitive High Piezoelectricity by Reentrant Relaxor Transition

**DOI:** 10.1002/advs.202508293

**Published:** 2025-07-17

**Authors:** Yang Yang, Shichang Li, Liqiang He, Guanqi Wang, Chang Liu, Yiqiao Song, Yuanchao Ji, Hanbing Zhang, Jiantuo Zhao, Dong Wang, Xiaobing Ren

**Affiliations:** ^1^ Frontier Institute of Science and Technology and State Key Laboratory for Mechanical Behavior of Materials Xi'an Jiaotong University Xi'an 710049 China; ^2^ Center for Advanced Smart Materials Yongjiang Laboratory Ningbo 315202 China

**Keywords:** (Ba,Ca)(Zr,Ti)TiO_3_, piezoelectric coefficient, reentrant relaxor transition, temperature stability

## Abstract

Although precision sensors and actuators demand piezoelectric materials with temperature‐insensitive high piezoelectricity (*d*_33_), achieving such a property is physically challenging because high *d*_33_ relies on tuning the material to the vicinity of a ferroelectric‐ferroelectric transition but it renders the property highly temperature‐sensitive. This issue is particularly prominent for lead‐free materials. Herein, a lead‐free Bi‐doped (Ba,Ca)(Zr,Ti)TiO_3_ ceramic showing a low‐temperature reentrant relaxor transition is designed, which is a diffuse transition from a tetragonal ferroelectric to a reentrant relaxor, the latter characterized by orthorhombic (O) polar nanodomains embedded in the tetragonal (T) ferroelectric matrix. The reentrant relaxor composition exhibits a remarkable temperature‐insensitive high *d*_33_ of ≈350 pC N^−1^ over a wide temperature range from ‐40 to 85 °C. In situ microscopic observations and phase field simulations reveal that the temperature‐insensitive high *d*_33_ in the reentrant relaxor composition originates from the synergistic effect between the reduction in the kinetic energy of the T‐symmetric ferroelectric domains and the increase in the volume fraction of the O‐symmetric nanodomains during cooling. This work provides a new recipe for designing lead‐free materials with temperature‐insensitive high piezoelectricity.

## Graphical abstract

Although precision sensors and actuators demand piezoelectric materials with temperature‐insensitive high piezoelectricity, achieving such a property is challenging. This paper reports that reentrant relaxor transitions can break the trade‐off between high piezoelectricity and temperature insensitivity. High piezoelectricity (350 pC N^−1^) is achieved in a lead‐free piezoelectric material that is temperature‐insensitive over a wide temperature range.

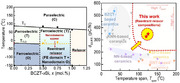

## 1 Introduction

Piezoelectric materials, which convert mechanical energy to electrical energy or vice versa, have been widely used in numerous electromechanical applications, ranging from piezoelectric sensors and actuators to medical ultrasound transducers and energy harvesters.^[^ ^1^, ^2^, ^3^, ^4^, ^5^ ^]^ The rapid advancement of smart technologies demands precision piezoelectric sensors and actuators that not only possess high sensing and actuating capabilities but also maintain such capability—quantified by the piezoelectric coefficient (*d*_33_)—insensitive to changes in operating temperature.^[^ ^6^, ^7^, ^8^, ^9^ ^]^ Consequently, these smart technology applications require piezoelectric materials with temperature‐insensitive high piezoelectricity (*d*_33_) over a wide temperature range (from cryogenic to high temperatures), especially lead‐free piezoelectrics, which are expected to serve as environmental‐friendly alternatives to toxic lead‐based materials.^[^ ^10^, ^11^ ^]^

To date, high piezoelectricity has typically been achieved by tuning the piezoelectric composition to the vicinity of a ferroelectric‐ferroelectric transition, known as a ferroelectric‐ferroelectric phase boundary in the phase diagram (e.g., morphotropic phase boundary (MPB) or polymorphic phase boundary (PPB)).^[^ ^11^, ^12^, ^13^, ^14^ ^]^ MPB or PPB facilitates polarization rotation, leading to a significant enhancement of *d*_33_.^[^ ^11^, ^13^, ^14^, ^15^ ^]^ However, in most lead‐free piezoelectric systems, this ferroelectric‐ferroelectric transition limits the high *d*_33_ to a narrow temperature range near the transition temperature, rendering the materials highly temperature sensitive.^[^ ^6^, ^7^, ^16^, ^17^, ^18^, ^19^ ^]^ Recent studied have reported textured ceramics and compositionally graded multilayer piezoelectric ceramics with temperature‐insensitive high *d*_33_ in the range of room temperature to 100 °C or 150 °C.^[^ ^6^, ^7^, ^20^, ^21^, ^22^, ^23^ ^]^ Whereas, their complex and costly fabrication processes make them unsuitable for large‐scale applications. Therefore, developing lead‐free piezoelectric ceramics with temperature‐insensitive high *d*_33_ over a wide temperature range, especially at low temperatures (the industrial requires a minimum operating temperature of ‐40 °C), remains a significant challenge. It is imperative to explore new strategies to address this critical issue.

In this work, a reentrant relaxor transition was introduced into the lead‐free (Ba,Ca)(Zr,Ti)TiO_3_ (BCZT) system through Bi^3+^ doping, resulting in compositions that exhibit remarkable temperature‐insensitive high *d*_33_ over a wide temperature range. The reentrant relaxor represents a unique state characterized by short‐range ordered nanodomains embedded in a long‐range ordered ferroelectric matrix, analogous to reentrant strain glasses in ferroelastic materials and reentrant spin glasses in ferromagnetic materials.^[^ ^16^, ^24^, ^25^, ^26^, ^27^, ^28^, ^29^ ^]^ Bi doping was selected because a small amount of Bi^3+^ doping can not only induce a diffuse phase transition, but also has little effect on the Curie temperature (*T*_C_) of BCZT materials.^[^ ^30^, ^31^, ^32^, ^33^ ^]^ Transmission electron microscopy (TEM) and phase field simulation results reveal that the reentrant relaxor composition undergoes a transition from a normal tetragonal ferroelectric phase to a microstructure consisting of orthorhombic polar nanodomains embedded within a matrix of tetragonal ferroelectric domains. This ferroelectric‐to‐reentrant relaxor transition not only yields a high *d*_33_ but also exhibits excellent temperature stability over a wide temperature range, thereby addressing the limitations of the conventional ferroelectric‐ferroelectric phase boundary mechanism. Our work provides a novel strategy for developing temperature‐insensitive high *d*_33_ lead‐free piezoelectric ceramics.

## 2 Results and Discussion

**Figure 1:**
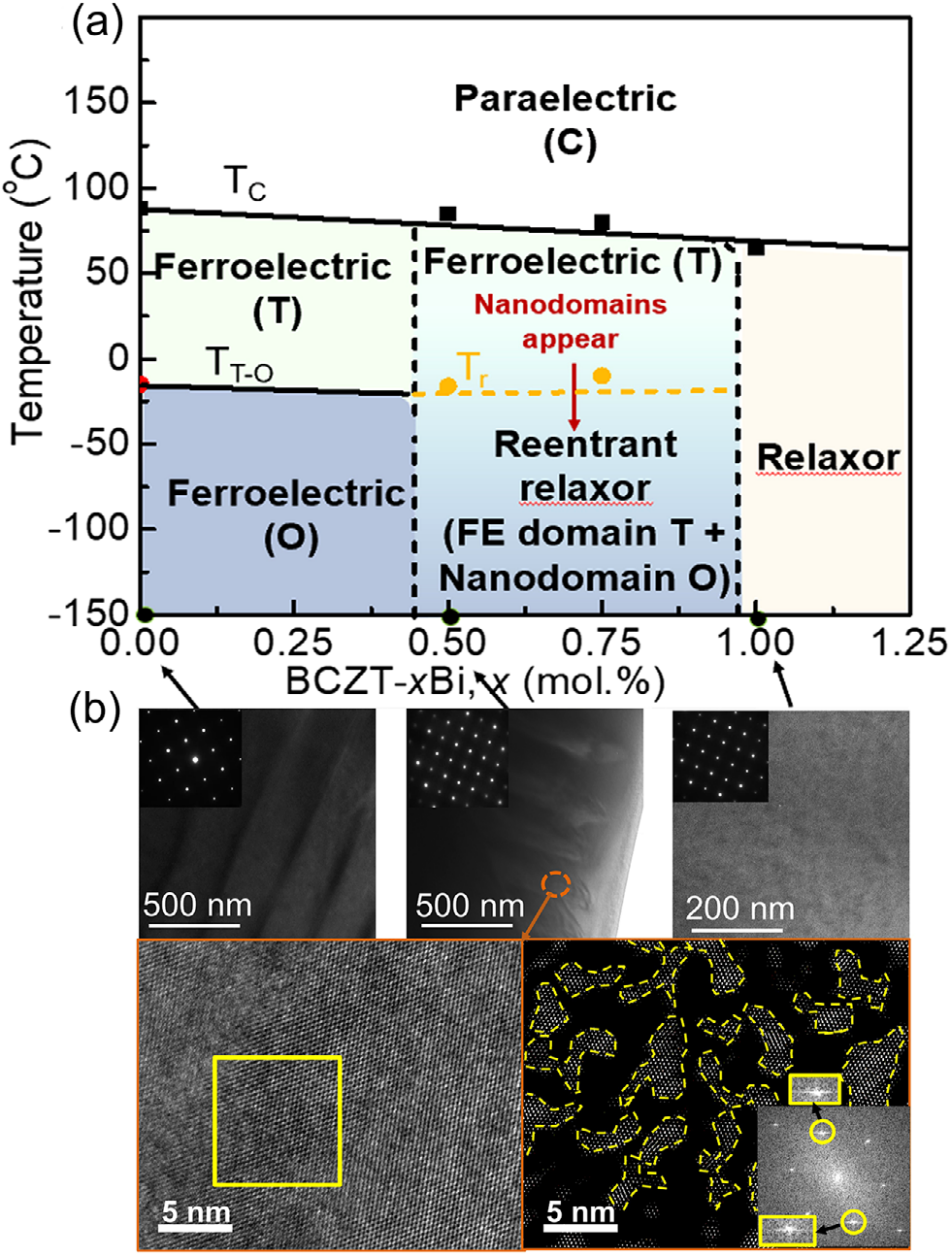
a) Phase diagram of BCZT‐*x*Bi ceramics. b) Bright‐field TEM images showing the microstructures of BCZT‐0Bi, BCZT‐0.5%Bi, and BCZT‐1%Bi ceramics. The high‐resolution TEM image and corresponding inverse fast Fourier transform (FFT) image of BCZT‐0.5%Bi ceramic reveal nanodomains (indicated by yellow dashed lines) with dimensions of approximately a few nanometers. The inset shows the FFT image, where elongated reflection spots (highlighted by yellow squares) arise from the formation of orthorhombic nanodomains.

**Figure 2:**
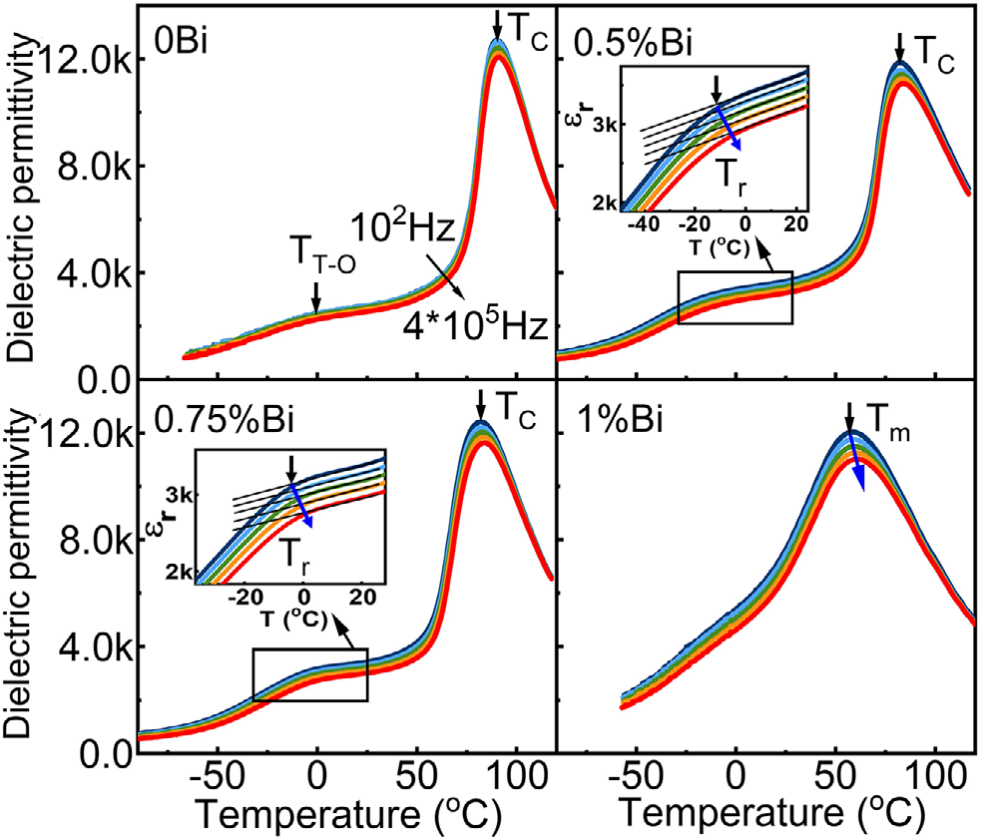
Temperature‐dependent dielectric permittivity of BCZT‐0Bi, BCZT‐0.5%Bi, BCZT‐0.75%Bi and BCZT‐1%Bi ceramics measured at various frequencies (100 Hz, 1000 Hz, 10,000 Hz, 100,000 Hz, 400,000 Hz) during cooling. The insets depict the frequency dispersion behavior of BCZT‐0.5%Bi and BCZT‐0.75%Bi ceramics around the reentrant relaxor transition temperature (*T*_r_).

**Figure 3:**
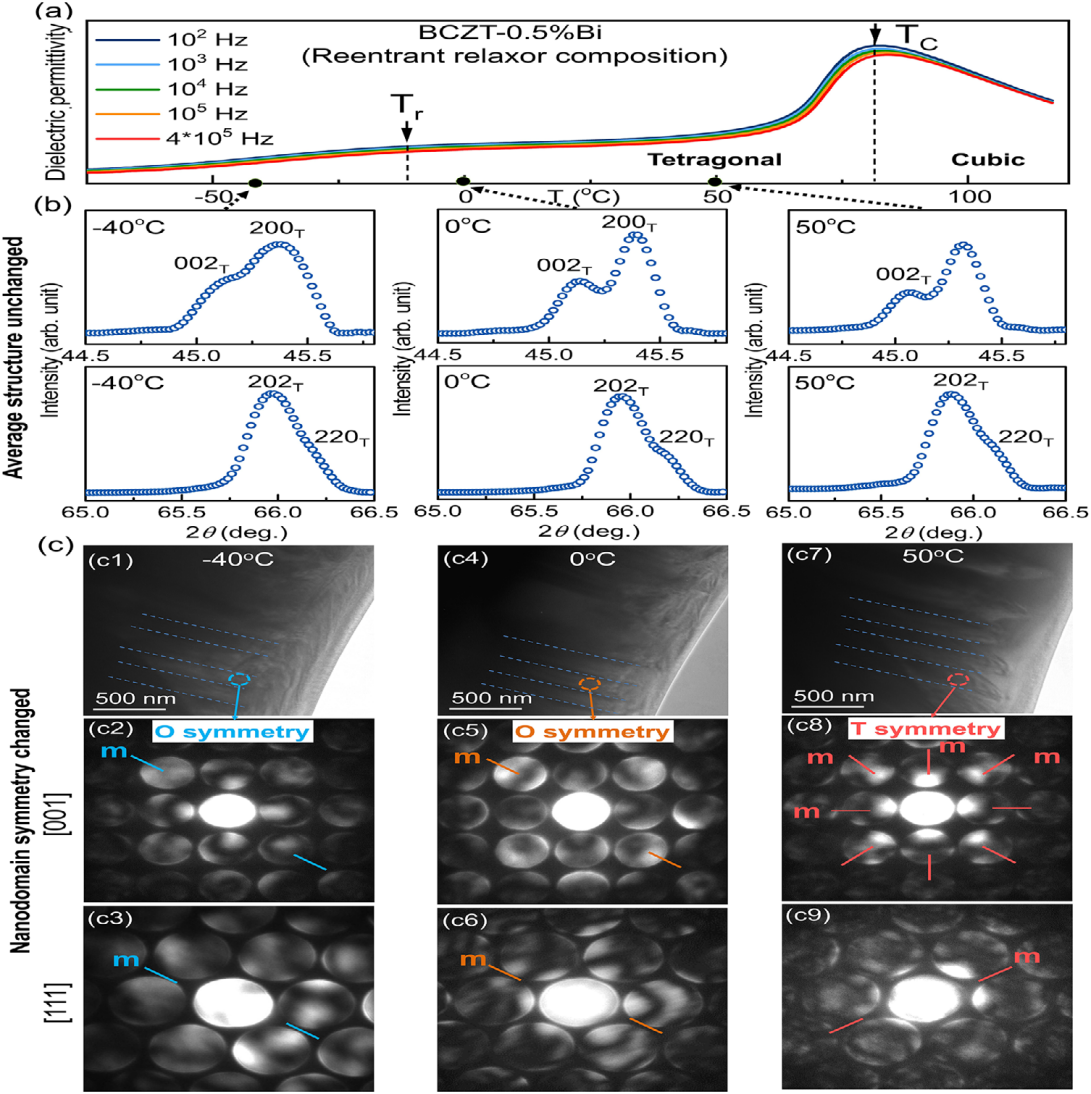
In situ XRD and microstructure analysis of BCZT‐0.5%Bi ceramic during cooling. a) Dielectric permittivity versus temperature curve of the sample. b) XRD profiles of BCZT‐0.5%Bi ceramic at 50 °C, 0 °C, and ‐40 °C. c) Bright‐field TEM images of BCZT‐0.5%Bi ceramic and the corresponding CBED patterns along the [001] and [111] zone axes. T and O denote tetragonal (*P4mm*) and orthorhombic (*Pm*) symmetries, respectively. Red, orange, and blue lines indicate the positions of the mirror planes.

All samples show a typical perovskite structure and high density, as confirmed by X‐ray diffraction (XRD) patterns and scanning electron microscope (SEM) micrographs provided in Figures S1 and S2 (Supporting Information). **Figure** **1**a illustrates the phase diagram of BCZT‐*x*Bi ceramics, constructed based on temperature‐dependent dielectric permittivity curves (**Figure** **2**) and transmission electron microscopy (TEM) results (Figure 1b and **Figure** **3**). The phase diagram is characterized by the existence of reentrant relaxor transitions, with BCZT‐0.5%Bi and BCZT‐0.75%Bi identified as reentrant relaxor compositions.

Bright‐field TEM images in Figure 1b depict the microstructures of BCZT‐0Bi (ferroelectric composition), BCZT‐0.5%Bi (reentrant relaxor composition), and BCZT‐1%Bi (relaxor composition) ceramics at room temperature. The BCZT‐0Bi sample exhibits conventional ferroelectric behavior, evidenced by the presence of large tetragonal ferroelectric domains. In contrast, the BCZT‐1%Bi sample displays typical relaxor characteristics, lacking large ferroelectric domains. The BCZT‐0.5%Bi sample, however, reveals a unique microstructure consisting of orthorhombic (O) polar nanodomains embedded within large tetragonal (T) ferroelectric domains. The existence of O‐symmetric nanodomains is demonstrated by the fast Fourier transform (FFT) and inverse FFT of the corresponding selected area in the high‐resolution TEM image, which shows elongated reflection spots and the presence of polar nanoregions. In situ XRD and convergent beam electron diffraction (CBED) patterns in Figure 3 further confirm that the reentrant relaxor composition exhibits an average tetragonal ferroelectric domain structure with localized O‐symmetric nanodomains.

Figure 2 presents the temperature‐dependent dielectric permittivity of BCZT‐*x*Bi ceramics measured during cooling at various frequencies, where *T*_C_ denotes the Curie temperature of the ferroelectric material, *T*_m_ represents the temperature of the dielectric maximum at 100 Hz, *T*_T‐O_ is the phase transition temperature from the tetragonal ferroelectric phase to the orthorhombic phase, and *T*_r_ is the reentrant relaxor transition temperature. At *T*_C_ or *T*_m_, all samples exhibit dielectric permittivity anomalies, corresponding to a phase transition from cubic to tetragonal symmetry during cooling. With increasing Bi content, *T*_C_ or *T*_m_ gradually decreases, accompanied by a broadening of the permittivity peak and more obvious frequency dispersion behavior. For the sample with 1% Bi content, a diffuse transition behavior with pronounced frequency dispersion is observed, indicating a state close to the relaxor state.^[^ ^34^, ^35^ ^]^ In contrast, samples with 0.5% and 0.75% Bi content first undergo a frequency‐independent paraelectric‐ferroelectric (cubic‐tetragonal, C‐T) transition, followed by a frequency‐dispersive transition upon cooling (Figure 2). The latter transition adheres to the Vogel‐Fulcher law (Figure S3, Supporting Information), showing the typical characteristics of a reentrant relaxor transition.^[^ ^25^, ^26^, ^27^, ^29^ ^]^

The *In‐situ* XRD patterns of the reentrant relaxor composition BCZT‐0.5%Bi are presented in Figure 3. From the dielectric permittivity versus temperature curve of BCZT‐0.5%Bi ceramic in Figure 3a, the sample at 50 °C corresponds to the tetragonal phase, while the sample at ‐40 °C represents another state after undergoing the reentrant relaxor transition. The XRD patterns of the BCZT‐0.5%Bi ceramic at 50 °C, 0 °C, and ‐40 °C all exhibit the same splitting of the 200 and 220 peaks, which is the characteristic of the average structure of the tetragonal phase, as shown in Figure 3b.

To further analyze the microstructure of the reentrant relaxor composition, in situ TEM and convergent beam electron diffraction (CBED) observations of BCZT‐0.5%Bi ceramic were measured at 50 °C, 0 °C, and ‐40 °C, as illustrated in Figure 3c. In BaTiO_3_‐based (BT) systems, the ferroelectric phase can possess *P4mm* tetragonal (T) symmetry, or *Pm* orthorhombic (O) symmetry. The former is characterized by four‐fold symmetry with four mirror planes along the [001] zone axis and one mirror plane along the [111] zone axis, while the latter possesses only one mirror plane along both the [001] and [111] zone axes.^[^ ^36^, ^37^, ^38^, ^39^ ^]^ At 50 °C, the bright‐field TEM image of BCZT‐0.5%Bi ceramic shows typical ferroelectric domains, and the corresponding CBED patterns confirm T symmetry. When the temperature drops to 0 °C and ‐40 °C (near or below the reentrant relaxor transition temperature), the large ferroelectric domains remain almost unchanged, consistent with the XRD results in Figure 3b and the TEM images in Figure S4 (Supporting Information). However, O‐symmetric polar nanoregions (PNRs) emerge within these large ferroelectric domains, as confirmed by the corresponding CBED patterns (Figure 3c) and high‐resolution TEM image (Figure 1b).

Combining the XRD patterns, HREM results, and CBED patterns, it is concluded that, unlike a normal ferroelectric transition, BCZT‐0.5%Bi ceramic presents tetragonal ferroelectric domains both before and after the reentrant relaxor transition. Near and after the transition, PNRs with O symmetry are embedded within the large T‐symmetric ferroelectric domains.

Figure S5 (Supporting Information) compares the structures and phase diagrams of the reentrant relaxor, canonical relaxor, and morphotropic phase boundary (MPB) compositions. Canonical relaxor ferroelectrics exhibit an average nonpolar cubic phase with the presence of polar nanoregions (PNRs), usually located on the higher doping concentration side of the phase diagram.^[^ ^34^, ^36^ ^]^ For MPB compositions, both the average structure and the microstructure exhibit the coexistence of multiple ferroelectric phases, being the boundary separating two different ferroelectric phases in the phase diagram.^[^ ^11^, ^40^ ^]^ In contrast, the reentrant relaxor maintains an average single ferroelectric phase before and after the reentrant transition,^[^ ^25^ ^]^ but its microstructure shows the coexistence of micrometer‐scale ferroelectric domains and nanodomains of different symmetries, which is usually located in the crossover region between the ferroelectric state and the relaxor state in the phase diagram. Consequently, the reentrant relaxor differs significantly from the canonical relaxors and MPB compositions in terms of average structure, microstructure, and phase diagram.

**Figure 4:**
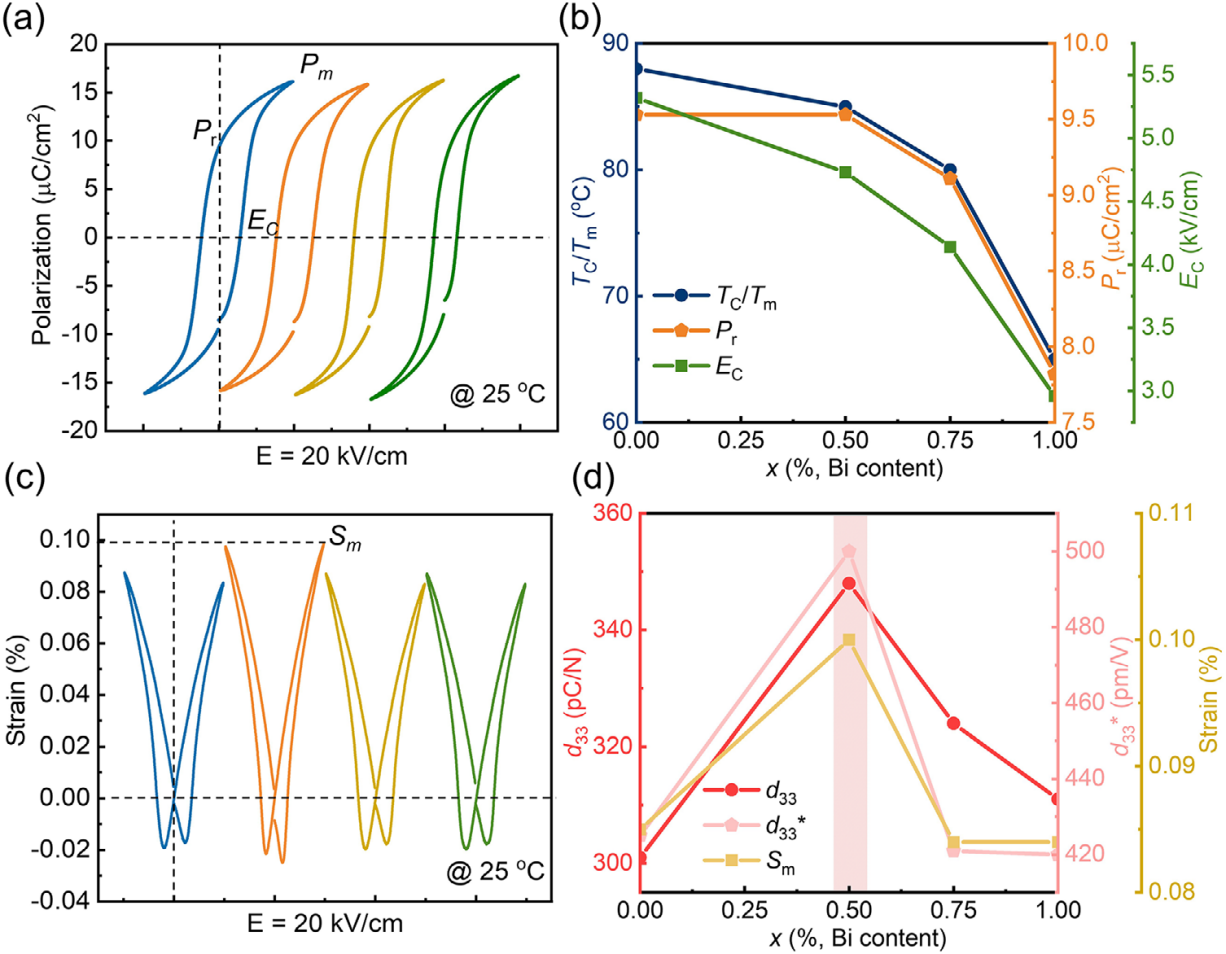
Room‐temperature polarization‐electric field (*P*‐*E*) loops a) and strain‐electric field (*S*‐*E*) loops c) of BCZT‐0Bi, BCZT‐0.5%Bi, BCZT‐0.75%Bi, and BCZT‐1%Bi ceramics measured under an electric field of 20 kV cm^−1^. b) Corresponding *T*_C_ or *T*_m_, remanent polarization (*P*_r_), and coercive field (*E*_C_) as a function of Bi content. d) Corresponding piezoelectric coefficient (*d*_33_), strain, and piezoelectric strain coefficient (*d*_33_^*^) as a function of Bi content, where *d*_33_^*^ is calculated as *S*_max_/*E*_max_.

The polarization‐electric field (*P*–*E*) hysteresis loops and bipolar strain–electric field (*S*–*E*) curves of BCZT‐0Bi, BCZT‐0.5%Bi, BCZT‐0.75%Bi and BCZT‐1%Bi ceramics were measured at room temperature (22 °C), as shown in **Figure** **4**a,c. While all samples exhibit typical ferroelectric hysteresis loops and butterfly‐shaped strain curves under an electric field of 20 kV cm^−1^, the *P*‐*E* hysteresis loops become slimmer with increasing Bi doping content, indicating an enhanced relaxation degree (also supported by dielectric permittivity and TEM results). This trend is particularly pronounced for the BCZT‐1%Bi ceramic, which can be seen more clearly in the composition dependence of *T*_C_ or *T*_m_, remnant polarization (*P*_r_), and coercive field (*E*_C_) curves (Figure 4b). The BCZT‐1%Bi ceramic exhibits the lowest *T*_m_, *P*_r_ and *E*_C_. Figure 4d shows the variations of the piezoelectric coefficient (*d*_33_), electrostrain, and piezoelectric strain coefficient (*d*_33_^*^) as a function of Bi doping content. The reentrant relaxor composition BCZT‐0.5%Bi exhibits significantly enhanced *d*_33_, strain and corresponding *d*_33_^*^. Details properties of each composition are listed in Table SI (Supporting Information).

Previous studies have demonstrated that the coexistence of multiple symmetric domains is beneficial to flatten the energy potential, thereby enhancing domain switching and improving dielectric and piezoelectric properties.^[^ ^41^, ^42^, ^43^ ^]^ Therefore, in the reentrant relaxor composition, the coexistence of ferroelectric domains and nanodomains with different symmetric leads to an enhancement of the piezoelectric coefficient d_33_. Notably, the *d*_33_ and high‐temperature (close to *T*_C_) stability of the BCZT‐0.75%Bi sample are slightly inferior to those of the BCZT‐0.5%Bi sample, which can be attributed to its proximity to the relaxor state and lower *T*_C_ (as shown in Figure 4b and Figure S6, Supporting Information). These characteristics lead to a more pronounced depolarization effect in the BCZT‐0.75%Bi sample, resulting in a non‐ideal poling state,^[^ ^44^ ^]^ especially in the high temperature region.

**Figure 5:**
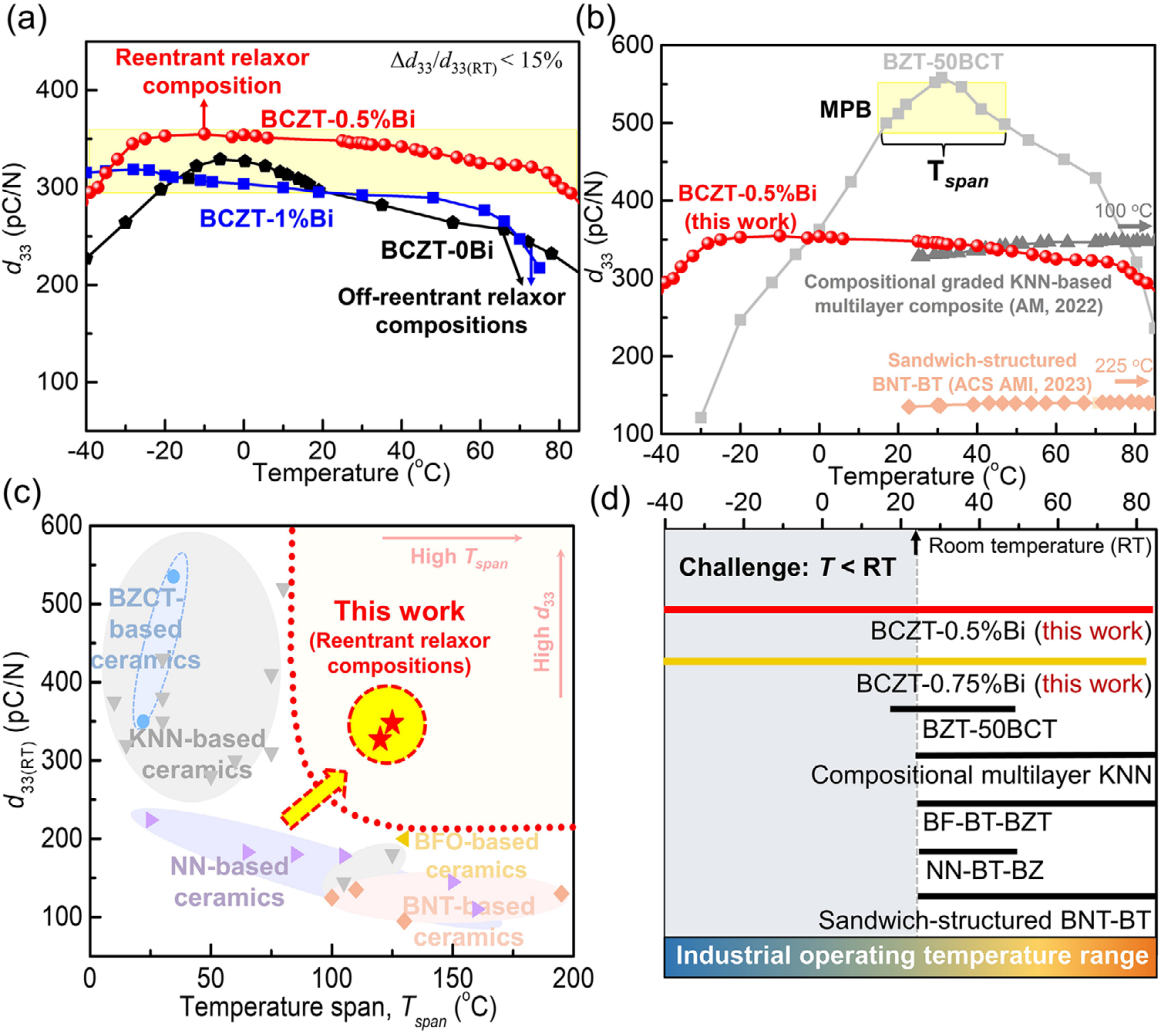
a) Temperature‐dependent *d*_33_ of BCZT‐0Bi, BCZT‐1%Bi, and BCZT‐0.5%Bi ceramics. b) Comparison of the temperature dependence of *d*_33_ for various lead‐free piezoelectric ceramics.^[^ ^6^, ^45^, ^50^ ^]^ c) *D*_33_ versus temperature span for reentrant relaxor ceramics and other lead‐free ceramics, where the temperature span is defined as the range over which the (*d*_33(_ *_T_*_)_‐*d*_33RT_)/*d*_33RT_ value is less than 15%.^[^ ^21^, ^45^, ^46^, ^47^, ^48^, ^49^, ^50^, ^51^, ^52^, ^53^, ^54^, ^55^, ^56^ ^]^ d) Comparison of the temperature‐insensitive *d*_33_ temperature span for various lead‐free piezoelectric ceramics.

Above we show that the reentrant relaxor composition exhibits a unique structure with an enhanced *d*_33_. Since the reentrant relaxor transition is inherently a diffuse transition, it is expected that the large *d*_33_ can be maintained over a wide temperature range. **Figure** **5**a shows the temperature‐dependent *d*_33_ of BCZT‐0Bi, BCZT‐0.5%Bi, and BCZT‐1%Bi ceramics measured within the industrial operating temperature range of ‐40 to 85 °C. The non‐reentrant relaxor transition compositions, BCZT‐0Bi and BCZT‐1%Bi, exhibit relatively low *d*_33_ and a moderate temperature‐insensitive range. The BCZT‐0Bi ceramic displays a *d*_33_ performance peak near the T‐O phase transition temperature. In contrast, the reentrant relaxor composition BCZT‐0.5%Bi demonstrates an enhanced *d*_33_ (≈350 pC N^−1^) and good temperature stability.

Figure 5b compares the temperature dependence of *d*_33_ for lead‐free Ba(Zr_0.2_Ti_0.8_)O_3_‐50(Ba_0.7_Ca_0.3_)TiO_3_ (BZT‐50BCT, a typical lead‐free piezoelectric ceramic with a ferroelectric‐ferroelectric phase boundary), BCZT‐0.5%Bi (the reentrant relaxor composition in this work), and other typical lead‐free (K,Na)NbO_3_‐based (KNN) and (Bi,Na)TiO_3_‐based (BNT) piezoelectric ceramics. The BZT‐50BCT ceramic shows a *d*_33_ performance peak around the ferroelectric‐ferroelectric phase transition temperature, which decreases sharply at low temperatures. Compositionally graded KNN‐based multilayer composites show a high *d*_33_ value of ≈330 pC N^−1^ and good temperature stability from room temperature to 100 °C.^[^ ^6^, ^7^ ^]^ Sandwich‐structured BNT‐BT ceramic shows a good temperature stability (above room temperature) but a low *d*_33_ value.^[^ ^45^ ^]^ In contrast, the reentrant relaxor composition BCZT‐0.5%Bi displays good temperature stability over a wide temperature range, particularly at low temperatures.

Figure 5c summarizes *d*_33_ versus temperature span for reentrant relaxor compositions in this work and other lead‐free (Ba,Ca)(Zr,Ti)O_3_‐based (BCZT‐based), KNN‐based, NaNbO_3_‐based (NN‐based), BNT‐based, and BiFeO_3_‐based (BFO‐based) ceramics, where the temperature span is defined as the range over which the (*d*_33(_ *_T_*_)_‐*d*_33RT_)/*d*_33RT_ value is less than 15%.^[^ ^21^, ^45^, ^46^, ^47^, ^48^, ^49^, ^50^, ^51^, ^52^, ^53^, ^54^, ^55^, ^56^ ^]^ The reentrant relaxor compositions BCZT‐0.5%Bi and BCZT‐0.75%Bi overcome the trade‐off between high *d*_33_ and temperature stability, exhibiting both high *d*_33_ and good temperature stability compared to other lead‐free piezoelectric ceramics. Figure 5d compares the *d*_33_‐insensitive temperature span of reentrant relaxor compositions with other lead‐free ferroelectric ceramics within the ambient operating temperature range.^[^ ^21^, ^45^, ^46^, ^47^, ^48^, ^49^, ^50^, ^51^, ^52^, ^53^, ^54^, ^55^, ^56^ ^]^ The reentrant relaxor ceramics exhibit large temperature spans and outperform other lead‐free ferroelectric materials, particularly in the low‐temperature window.

Additionally, the aging and cycling stability tests of the BCZT‐0.5%Bi ceramic (detailed testing conditions and results are provided in Figure S7, Supporting Information) confirm the excellent stability of its high d_33_ performance. These results suggest that reentrant relaxor materials hold significant promise for practical applications. However, it is worth noting that although the materials developed in this work exhibits temperature‐insensitive high *d*_33_ over a broad temperature range, their relatively low *T*_C_ limits their applications at high temperatures. Therefore, such materials are more suitable for medical detection and therapy, as well as scientific exploration in polar regions and other low‐temperature environments.^[^ ^57^ ^]^ To increase the *T*_C_ of reentrant relaxor compositions while maintaining high *d*_33_ and temperature stability, our future research will further focus on co‐doping, sintering process optimization, and the introduction of reentrant relaxor transitions into high *T*_C_ systems.

**Figure 6:**
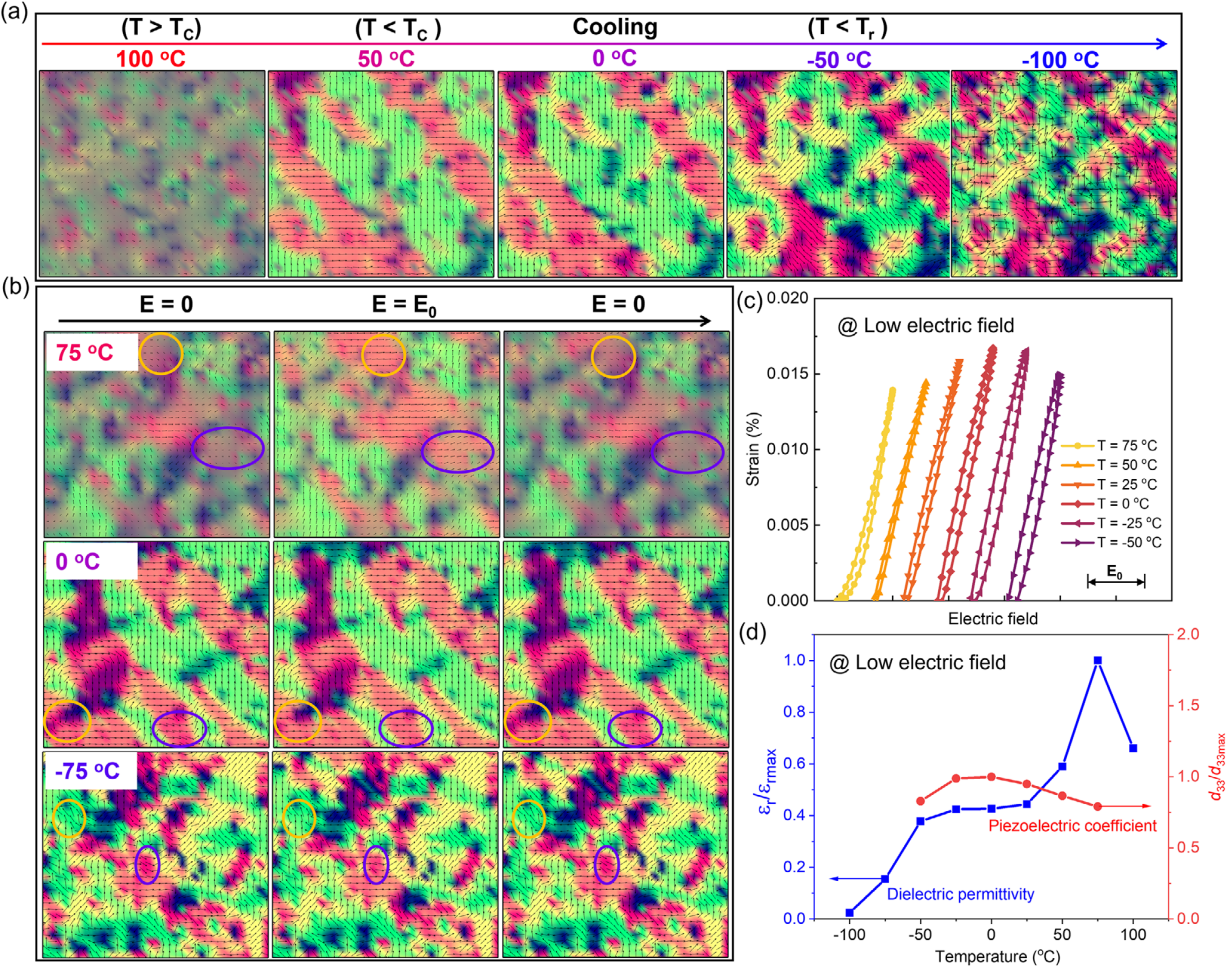
a) Calculated microstructural evolution of the reentrant relaxor composition from high temperature (above *T*_C_) to low temperature. Different colors and arrows represent distinct polarization domains. b) Microstructural evolution of the reentrant relaxor composition at different temperatures under loading and unloading conditions at low electric field *E*_0_ (*E*_0_ << *E*_C_). c) Calculated ultralow electric field‐induced strain curves at different temperatures. d) Calculated temperature‐dependent dielectric permittivity and *d*_33_ curves of the reentrant relaxor sample.

To elucidate the microstructural evolution upon cooling (or under an electric field) and the underlying mechanism of the reentrant relaxor composition yielding temperature‐insensitive high *d*_33_ over a wide temperature range, phase‐field simulations were performed on the reentrant realxor BCZT‐0.5%Bi ceramic. **Figure** **6**a illustrates the microstructural evolution of the reentrant relaxor composition upon cooling from above the Curie temperature (*T*_C_) to low temperatures. At temperatures above *T*_C_ (100 °C), the sample is in the paraelectric state. When the temperature drops below *T*_C_, the sample undergoes a paraelectric‐to‐ferroelectric phase transition, forming large T‐symmetric ferroelectric domains. Further cooling leads to the emergence of O‐symmetric polar nanodomains embedded within the T‐symmetric ferroelectric matrix, resulting in the coexistence of O‐symmetric nanodomains and T‐symmetric ferroelectric domains over a wide temperature range. Figure S8 (Supporting Information) shows the quantitative volume fraction of O‐symmetric nanodomains in the BCZT‐0.5%Bi composition as a function of temperature, revealing that the volume fraction of O‐symmetric nanodomains increases with decreasing temperature.

In this work, material defects arise from the high concentration of point defects incorporated into the perovskite structure, leading to an increase in lattice defects. Due to factors such as ionic radius mismatch, these lattice defects induce lattice distortion and generate randomly distributed internal stress fields. Previous studies have not fully explained why defects do not suppress the first paraelectric‐to‐ferroelectric phase transition but instead suppress subsequent phase transition.^[^ ^24^, ^25^, ^28^, ^58^ ^]^ The phase‐field simulations in this work propose a potential explanation for the origin of the reentrant relaxor transition. During cooling, although the concentration of point defects remains constant, the stress field generated by these defects intensifies as the temperature decreases.^[^ ^59^ ^]^ At relatively high temperatures near *T*_C_, the stress field is insufficient to suppress the C‐T phase transition. However, as the temperature decreases further, the enhanced stress field effectively suppresses the subsequent T‐O phase transition, resulting in the formation of O‐symmetric nanodomains. The simulated microstructural evolution aligns well with experimental observations.

Figure 6b depicts the microstructural evolution of the reentrant relaxor composition at different temperatures under loading and unloading conditions at low electric field *E*_0_ (*E*_0_ << *E*_C_). Based on the simulated microstructural evolution, the temperature‐dependent dielectric permittivity curves and ultralow electric field (*E*<<*E*_C_)‐induced strain curves were calculated, as shown in Figure 6c,d, respectively. These results are in good agreement with experimental data. The *d*_33_ calculated from the low electric field‐induced strain curves (Figure 6c) shows good temperature stability, even at low temperatures. The temperature‐insensitive high *d*_33_ in the reentrant relaxor composition primarily arises from the interplay between two competing factors: (1) the downward trend in the piezoelectric response caused by the reduction in the kinetic energy of T‐symmetric ferroelectric domains during cooling, and (2) the upward trend in the piezoelectric response due to the increasing volume fraction of O‐symmetric nanodomains during cooling. Specifically, the stabilization of ferroelectric domains at low temperatures reduces their responsiveness to small‐field stimulation, leads to a decrease in *d*_33_. This phenomenon is exemplified by the BZT‐50BCT piezoelectric ceramic, which exhibits a single‐phase ferroelectric state below room temperature; its *d*_33_ decreases markedly as the temperature drops from room temperature to low temperatures (Figure 5b). However, reentrant relaxor materials exhibit a coexisting structure of ferroelectric domains and nanodomains with different symmetries. During cooling, the volume fraction of O‐symmetric nanodomains increases, leading to changes in polarization and an increase in *d*_33_. The competition between these two factors enables the material to maintain temperature‐insensitive high *d*_33_ over a wide temperature range. This work provides new insights into the design of temperature‐insensitive high‐*d*_33_ lead‐free piezoelectric ceramics.

## 3 Conclusion

By introducing the reentrant relaxor transition into the lead‐free Bi‐modified BCZT system, enhanced piezoelectric coefficient and good temperature stability down to low temperatures are achieved in reentrant relaxor ceramics. In situ microscopic observations and phase‐field simulations reveal that the temperature‐insensitive high piezoelectric response in the reentrant relaxor composition originates from the interplay between two competing factors: (1) the downward trend in the piezoelectric response caused by the reduction in the kinetic energy of T‐symmetric ferroelectric domains during cooling, and (2) the upward trend in the piezoelectric response caused by the increasing volume fraction of O‐symmetric nanodomains during cooling. The elucidation of the microscopic nature of reentrant relaxor transitions provides new insights into their potential technological applications. This work provides a new approach to developing lead‐free piezoelectric ceramics with high piezoelectric coefficients and excellent temperature stability over a wide temperature range.

## 4 Experimental Section

### Sample Preparation

Ba_0.82‐3_ *_x_*_/2_Ca_0.18_Zr_0.11_Ti_0.89_O_3_‐*x*Bi (0 ≤ *x* ≤ 1%) (BCZT‐*x*Bi) ceramics were fabricated by a conventional solid‐state method. Pre‐dried raw materials, including BaCO_3_ (99.8%), TiO_2_ (99.8%), CaCO_3_ (99.9%), ZrO_2_ (99.8%), and Bi_2_O_3_ (99.99%), were mixed with anhydrous ethanol in a nylon jar and ball‐milled for 12 h using a planetary mill. The mixed powder was dried and calcined in an alumina crucible at 1300 °C for 3 h. The calcined powder was ball‐milled again for 12 h, dried at 90 °C, and then pressed into pellets with a diameter of 8 mm. The pellets were sintered in air at 1480 °C for 4 h. Ba(Zr_0.2_Ti_0.8_)O_3_‐50(Ba_0.7_Ca_0.3_)TiO_3_ (BZT‐50BCT) ceramics were prepared using the same method. To establish a low‐temperature reentrant relaxor transition and achieve high *d*_33_ in a wide temperature range, zirconium (Zr) and calcium (Ca) contents in the BCZT‐*x*Bi compositions were adjusted based on previously reported BZT‐50BCT ceramics.

### Structure and Electrical Property Characterization

The phase structure of the samples was determined using an X‐ray diffractometer (XRD, Shimadzu 7000). Grain morphologies were observed using a field emission scanning electron microscope (SEM; JSM‐5610, JEOL, Tokyo, Japan). Dielectric permittivity versus temperature was measured using a HIOKI LCR meter at various frequencies with a cooling rate of 2 °C min^−1^. Ferroelectric and strain measurements, including electric‐field induced polarization (*P*‐*E*) hysteresis loops and unipolar/bipolar strain (*S*‐*E*) loops, were performed using a ferroelectric tester system (Radiant Workstation, Radiant and SIOS‐SP‐S120). Samples with silver electrodes were poled under a DC electric field of 1.5 kV mm^−1^ for 30 min at room temperature. The temperature dependence of the piezoelectric coefficient *d*_33_ was measured using a piezoelectric *d*_33_‐meter (Model ZJ‐3A, Chinese Academy of Sciences) with a self‐made temperature chamber at a cooling/heating rate of ≈10 °C min^−1^ (heating above room temperature and cooling below room temperature). Transmission electron microscopy (TEM) and convergent beam electron diffraction (CBED) were performed using a JEOL‐2100F equipped with a double‐tilt specimen stage.

### Phase‐Field Simulations

Phase field simulations were employed to elucidate the microstructural evolution and related properties of a model system exhibiting cubic (C) to tetragonal (T) to orthorhombic (O) transitions, incorporating the local field effect caused by defects. The total free energy includes the following terms:

(1) $$F=\int_{V}f_{bulk}+f_{local}+\int_{V}\left(f_{elas}+f_{elec}+f_{grad}\right)\;dV$$

(2) $$\begin{array}{cc} f_{bulk} & =A_{1}\;\left(P_{1}^{2}+P_{2}^{2}+P_{3}^{2}\right)-A_{2}{\left(P_{1}^{2}+P_{2}^{2}+P_{3}^{2}\right)}^{2}\\ & \;+A_{12}\left(P_{1}^{2}P_{2}^{2}+P_{2}^{2}P_{3}^{2}+P_{1}^{2}P_{3}^{2}\right)\\ & \;+A_{13}\left(P_{1}^{4}P_{2}^{2}+P_{2}^{4}P_{3}^{2}+P_{1}^{4}P_{3}^{2}+P_{1}^{2}P_{2}^{4}+P_{2}^{2}P_{3}^{4}+P_{1}^{2}P_{3}^{4}\right)\\ & \;+A_{14}\left(P_{1}^{2}P_{2}^{2}P_{3}^{2}\right)+A_{3}{\left(P_{1}^{2}+P_{2}^{2}+P_{3}^{2}\right)}^{3}\\ & \;+A_{15}\left(P_{1}^{6}P_{2}^{2}+P_{2}^{6}P_{3}^{2}+P_{1}^{6}P_{3}^{2}+P_{1}^{2}P_{2}^{6}+P_{2}^{2}P_{3}^{6}+P_{1}^{2}P_{3}^{6}\right) \end{array}$$

The term *f*_bulk_ in Equation (1) describes the bulk free energy density, which can be expressed by Landau free energy term in Equation (2) from the previous study.^[^ ^60^, ^61^, ^62^ ^]^ It should be noticed that the multiply terms (*A*_12_, *A*_13_, *A*_14_) in Equation (2) decide the stability of ferroelectric phases (T, O, or R) and transition sequence. The dimensionless coefficients *A*_1_ = 0.00412 × (*T* + 2500*x –* 80), *A*_2_ = 2.0, *A*_12_ = ‐4.0 + 5 × (*T* + 50) / 50, *A*_14_ = 77.64, *A*_3_ = 12.94, *A*_15_ = 492. The term *f*_local_ represents the energy density due to the local electric field *E*_local_ caused by doping,^[^ ^63^ ^]^ which exhibits temperature dependence (*E*_local_ = *E* ^0^ × 0.014 × exp(1000 / *T*)) in our simulations. The terms *f*_elas_, *f*_elec_ and *f*_grad_ in Equation (1) reflect the long‐range elastic and electrostatic interaction energies and the short‐range exchange interaction energy of the gradient term, respectively. The term *f*_elec_ = *f*_dipole_ + *f*_depola_ + *f_appl,_* ^[^ ^64^, ^65^ ^]^ where *f*_dipole_ is the dipole‐dipole interaction caused by polarization, *f*_depola_ is the depolarization energy density, and *f_appl_* is the energy density caused by the applied electric field. The temporal evolution of the spontaneous polarization field is obtained by solving the time‐dependent Ginzburg‐Landau (TDGL) function as Equation (3):

(3) $$\frac{dP_{i}\left(x,t\right)}{dt}=-M\frac{\delta F}{\delta P_{i}\left(x,t\right)},i=1,2,3$$

The domain structure is described by the distribution of spontaneous polarization *P* = (*P*_1_, *P*_2_, *P*_3_). Further mathematical transformations were employed to obtain the symmetry contours from the calculated vector maps.

## Conflict of Interest

The authors declare no conflict of interest.

## Supporting information



Supporting Information

## Data Availability

The data that support the findings of this study are available from the corresponding author upon reasonable request.
