# Supplementary material for: Achieving Temperature‐Insensitive High Piezoelectricity by Reentrant Relaxor Transition
Source: Adv Sci (Weinh). 2025 Jul 17;12(38):e08293. doi: 10.1002/advs.202508293 (PMC12520457; doi:10.1002/advs.202508293)
Supplement: Supplementary file 1 — Supporting Information [file ADVS-12-e08293-s001.pdf]

ADVANCED SCIENCE
Open Access

Supporting Information

for *Adv. Sci.*, DOI 10.1002/advs.202508293

Achieving Temperature-Insensitive High Piezoelectricity by Reentrant Relaxor Transition

Yang Yang, Shichang Li, Liqiang He, Guanqi Wang, Chang Liu, Yiqiao Song, Yuanchao Ji*, Hanbing Zhang, Jiantuo Zhao, Dong Wang* and Xiaobing Ren**

Supporting Information

Achieving temperature-insensitive high piezoelectricity by reentrant relaxor transition

Yang Yang[1*], Shichang Li[1], Liqiang He[1], Guanqi Wang[1], Chang Liu[1], Yiqiao Song[1], Yuanchao Ji[1*], Hanbing Zhang[1], Jiantuo Zhao[1], Dong Wang[1*], Xiaobing Ren[1, 2*]

[1]*Frontier Institute of Science and Technology, and State Key Laboratory for Mechanical Behavior of Materials, Xi'an Jiaotong University, Xi'an 710049, China*

[2]*Center for Advanced Smart Materials, Yongjiang Laboratory, Ningbo 315202, China*

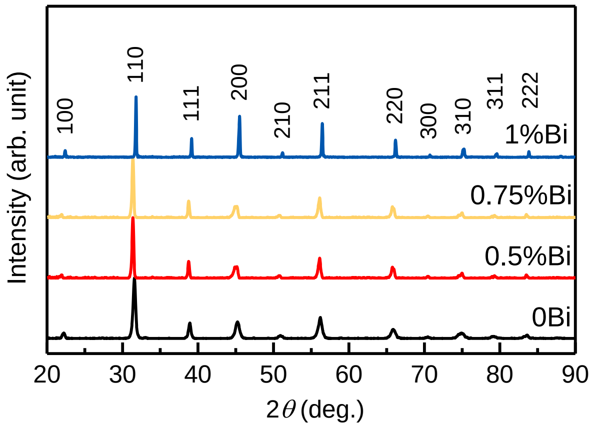

Figure S1. XRD patterns of BCZT-0Bi, BCZT-0.5%Bi, BCZT-0.75%Bi and BCZT-1%Bi ceramics measured at room temperature in the range of 2θ = 20-90°.

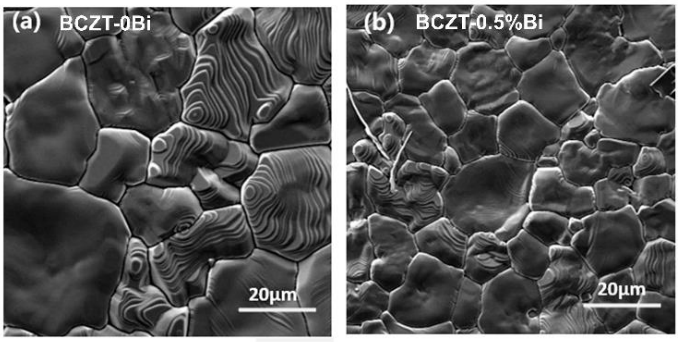

Figure S2. Room temperature SEM micrographs of (a) BCZT-0Bi and (b) BCZT-0.5%Bi ceramics.

Figure S3 shows the peak temperature of the dielectric permittivity of BCZT-*x*Bi ceramics as a function of the Napierian logarithm of the test frequency. The frequency/temperature characteristics of the sample can be well fitted by the Vogel-

Fulcher relationship as shown below:

$$f = f_0 \exp(-\frac{U}{T - T_f}) \Rightarrow \ln f = \ln f_0 + \frac{-U}{T + (-T_f)} \tag{1}$$

where：

f —— Test frequency/Hz;

f_0—— Debye frequency/Hz;

U —— Average activation energy/J;

T —— Peak temperature corresponding to each test frequency/ °C;

T_f ——Static freezing temperature/ °C.

After fitting the data extracted from the dielectric response according to the Vogel-Fulcher relation, we obtained the curves shown in Figure S3.

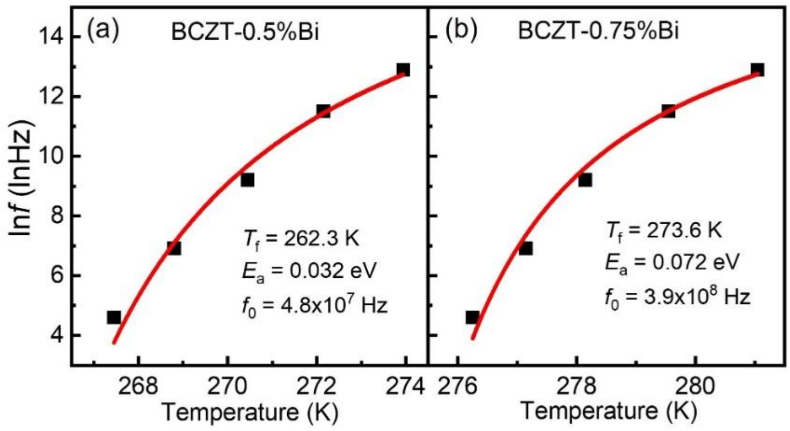

Figure S3. Dielectric permittivity peak temperature as a function of the Napierian logarithm of the test frequency of BCZT-*x*Bi ceramics, and the line is the Vogel-Fulcher fitting curve.

Figure S4 shows the *in situ* TEM results of BZT-50BCT (MPB composition) and BCZT-0.5%Bi (reentrant relaxor composition) ceramics. As can be seen, in the typical lead-free ferroelectric-ferroelectric phase boundary case (i.e., MPB), the domain structure of BZT-50BCT ceramic changes with the temperature from 25 °C drops to -40 °C. Therefore, it is reasonable that the d_{33} changes as the temperature decreases due to the variation of domain structure and stabilization of the ferroelectric domains.

However, the reentrant relaxor BCZT-0.5%Bi ceramic shows a stable domain structure as the temperature decreases (Figure S4(b1-b2)).

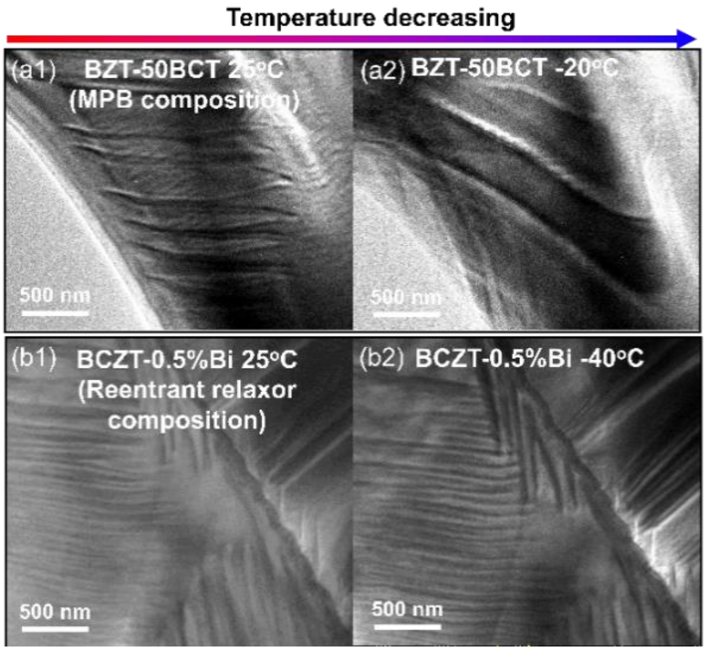

Figure S4. (a1-a2) *In situ* microstructure evolution of MPB composition BZT-50BCT ceramic at 25 °C (at MPB) and -20 °C (below MPB). (b1-b2) *In situ* microstructure evolution of ferroelectric-ferroelectric phase composition BCZT-0.5%Bi ceramic at 25 °C and -40 °C.

Table I summarizes the different properties of each composition, including data obtained through Vogel-Fulcher (V-F) curves.

Table SI. Summary of properties of BCZT-xBi ceramics

Composition	ε_{rRT} (10^3Hz)	T_C/ T_m, °C	d_{33RT}, PC/N	E_C, kV/cm	T_{f2}, °C	E_{a2}, eV	f_{02}, Hz
0Bi	2805	88	301	5.32			
0.5%Bi	2907	85	349	4.73	-11.5	0.032	4.8×10^7
0.75%Bi	3500	80	324	4.14	0.1	0.072	3.9×10^8
1%Bi	7485	65	311	2.96			

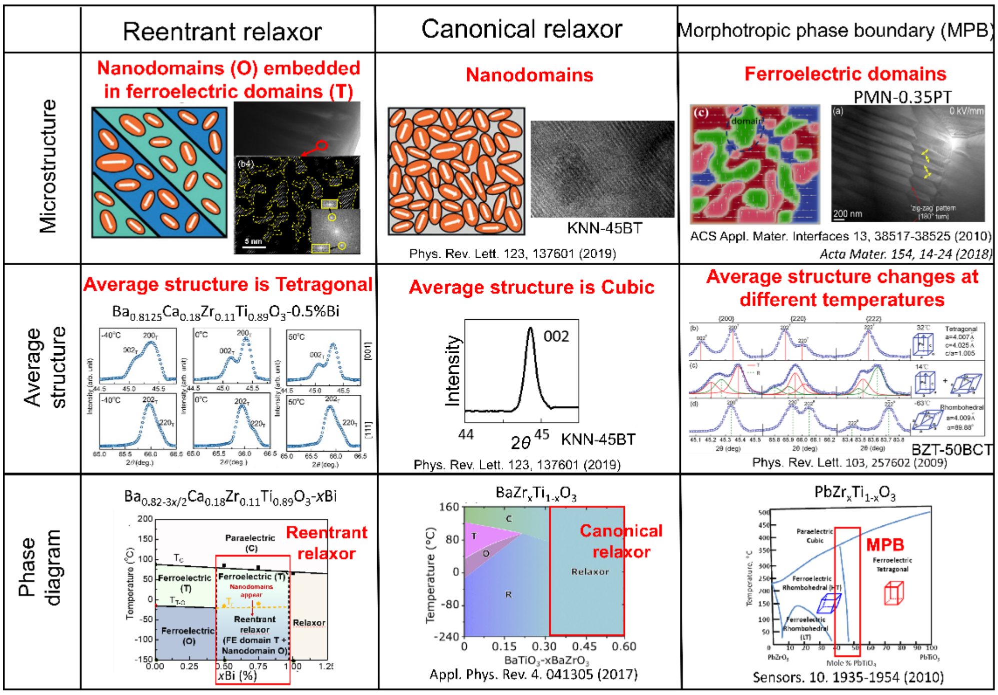

Figure S5. Comparison of the reentrant relaxor, canonical relaxor and morphotropic phase boundary in structures and phase diagrams.

The dispersion coefficient γ of $Ba(Zr_{0.2}Ti_{0.8})O_3$-50$(Ba_{0.7}Ca_{0.3})TiO_3$ (BZT-50BCT), BCZT-0.5%Bi and BCZT-0.75%Bi ceramics had been calculated using the modified Curie-Weiss law: $1/\varepsilon - 1/\varepsilon_{max} = (T - T_m)^{\gamma}/C$, where ε_{max} is the maximum value of the permittivity; T_m is the corresponding temperature; C and γ are assumed to be constant, and γ gives information about the dispersion degree of the samples. The plots of $\ln(1/\varepsilon - 1/\varepsilon_{max})$ as a function of $\ln(T\text{-}T_m)$ are shown in Figure S6. The γ fitting value of BZT-50BCT ceramic is 1.56. And the γ fitting value of BCZT-0.5%Bi ceramic is 1.74, the γ fitting value of BCZT-0.75%Bi ceramic reaches 1.77.

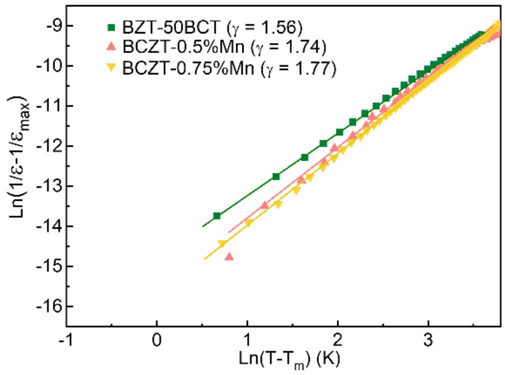

Figure S6. Plot of $\ln(1/\varepsilon - 1/\varepsilon_{max})$ as a function of $\ln(T\text{-}T_m)$ for BZT-50BCT, BCZT-0.5%Bi, and BCZT-0.75%Bi ceramics.

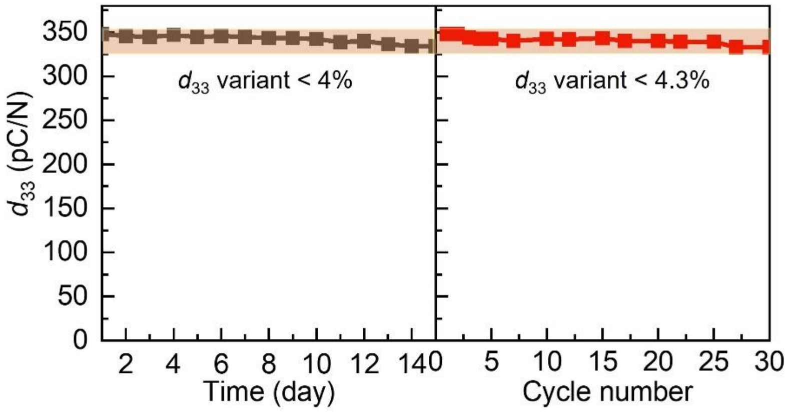

Figure S7. Variation of d_{33} of the BZCT-0.5%Bi ceramic with time (aging at room temperature for 1–15 days) and cycle number. For the cycling stability test, the sample was first annealed at 0 °C for 5 min, then annealed at 40 °C for 5 min, followed by measuring the d_{33} value at room temperature.

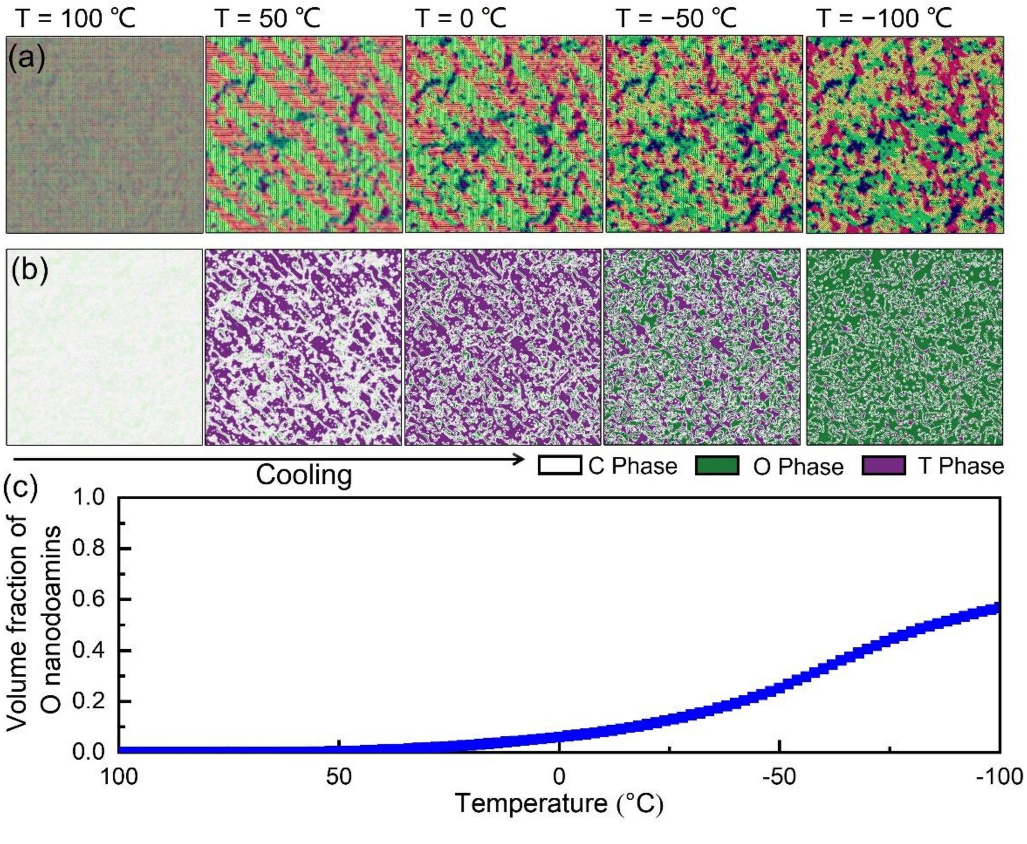

Figure S8. Microstructural evolution with upon cooling (with expanded domain range). (a) Polarization vector field, with arrows indicating components along P_x, P_y, and P_z. (b) Phase contour showing the C (white), T (purple), and O (green) phases. (c) The temperature-dependent variation of the volume fraction of O nanodomains in the BCZT-0.5%Bi sample.
